# 

**Published:** 1896-02-29

**Authors:** 


### MEDICINE AT OXFORD.

It appears likely that a statute will be passed at the University of Oxford which will bring its medical faculty and the Radcliffe Infirmary into closer relationship. This statute provides for the pathological department of the infirmary being placed under the Regius Professor of Medicine. We hope this movement will be the beginning of better days for the infirmary, and that the University will feel a greater interest in the institution henceforth.

**Notices of Births, Deaths, and Marriages** are inserted in this column at a charge of 2s. 6d. for an announcement not exceeding four lines.

**Notice to Advertisers and Subscribers.**

All Advertisements, Orders for Copies of The Hospital and Business Communications should be addressed to **THE MANAGER, (Not to The Editor),** The Hospital, 428, Strand, London, W.C.

The Cost of Subscription, post free, is as follows:—Per week, 2½d.; per quarter, 2s. 8d.; per half-year, 5s. 3d.; per annum, 10s. 6d. Foreign:—Per Annum, 15s. 2d.; payable, in all cases, in advance.

### NOTICE TO CORRESPONDENTS.

All MS., Letters, Books for Review, and other matters intended for the Editor should be addressed The Editor, The Lodge, Porchester Square, London, W.

The Editor cannot undertake to return rejected MS., even when accompanied by stamped directed envelope.

**"Burdett's Hospitals and Charities."**

Sixth year of publication. 950 pp. Scarlet cloth, gilt lettered, Price 5s. A most complete guide to British, Colonial, Indian, and American Hospitals, Dispensaries, Nursing and Convalescent Institutions, and Asylums. A few complete sets of the preceding five issues of the "Annual" may still be obtained on application to the Publishers, The Scientific Press (Limited), 428, Strand, London, W.C.

**NOTICE.—Vol. XVIII. of "The Hospital" (April to September, 1895), in handsome cloth binding, is on sale at the Office of this Journal. Price 7s. 6d. Cases for binding the Numbers contained in this Volume, 1s. 6d. Index to Vol. XVIII., 2½d., post free. The Monthly Part for March will be ready next week, price 6d., by post, 8d.**

### Books Received.

"Christian Globe" Office.
"Earthly Footsteps of the Man of Galilee."

A. and C. Black.
"Text Book of General Pathology and Pathological Anatomy." By Richard Thoma. Translated by Alexander Bruce, M.A., M.D. Volume I.

Longmans, Green, and Co.

"Stray Thoughts for Invalids." By Lucy H. M. Soulsby.

William Reeves.
No. 7 the Humanitarian League's publications, "I Was in Prison."

Kelly and Co.
"Kelly's London Medical Directory, 1896."

W. H. and L. Collingridge.

"Encyclopædia of Gardening." By T. W. Sanders, F.R.H.S.

**Periodicals and Pamphlets.**—Child's Guardian, The Planter, Toilers of the Deep, Medical Week, British and Colonial Druggist, Medical Press, Charity Record, Edinburgh Medical Journal, The Sanitary Record, London, Humanitarian, Windsor Magazine, Practitioner, International Medical Magazine, Homœopathic World, Leeds Hospital Magazine, First Aid, Dagny, Invention, Monthly Homœopathic Review, Sixth Annual Report of the Vegetarian Federal Union, Literary Digest, New York Medical Journal, Columbus Medical Journal, Clinical Journal, Westminster Review, Electrical Review, Edinburgh Medical Missionary Society Quarterly Paper, Glasgow Medical Journal, Charlotte Medical Journal, Children's League of Pity Paper, Pediatrics, Tri-State Medical Journal, Medical Pioneer, Vectis.

### THE STUDENT OF MEDICINE.

### APPOINTMENTS.

St. Thomas's Hospital House Appointments.—The following gentlemen have been selected as house officers from Tuesday, March 3rd, 1896: Resident house physicians, F. B. Thornton, L.R.C.P., M.R.C.S. (extension), and W. E. Dixon, B.Sc.Lond., L.R.C.P., M.R.C.S. (extension); non-resident house physicians, E. W. Palin, M.A., M.B., B.Ch.Oxon, L.R.C.P., M.R.C.S., and P. S. Hichens, M.A., M.B., B.Ch.Oxon, L.R.C.P., M.R.C.S.; house surgeons, L. A. R. Wallace, B.A., M.B., B.Ch.Oxon, L.R.C.P., M.R.C.S., H. C. Crouch, L.R.C.P., M.R.C.S., J. L. Prain, L.R.C.P., M.R.C.S., and G. J. Conford, B.A., M.B., B.Ch.Oxon, L.R.C.P., M.R.C.S.; assistant house surgeons, B. Dyball, L.R.C.P., M.R.C.S., P. W. Kent, L.R.C.P., M.R.C.S., J. Smith, B.A., M.B., B.C.Cantab, L.R.C.P., M.R.C.S., and W. D. Frazer, L.R.C.P., M.R.C.S.; obstetric house physicians, senior, G. G. Genge, L.R.C.P., M.R.C.S., and junior, C. W. Grant Wilson, L.R.C.P., M.R.C.S.; ophthalmic house surgeons, senior, A. H. P. Dawnay, L.R.C.P., M.R.C.S., and junior, E. A. Saunders, M.A., M.B., B.Ch.Oxon, L.R.C.P., M.R.C.S.; clinical assistants in the special departments for diseases of the throat, W. Thornely, B.A.Cantab, L.R.C.P., M.R.C.S. (extension), and P. L. Blaber, L.R.C.P., M.R.C.S.; clinical assistants in the special departments for diseases of the skin, W. D. Knocker, L.R.C.P., M.R.C.S. (extension), and G. R. Harcourt, L.R.C.P., M.R.C.S. (extension); clinical assistants in the special departments for diseases of the ear, W. H. J. Paterson, L.R.C.P., M.R.C.S., and R. G. Strange, L.R.C.P, M.R.C.S.; clinical assistants in the electrical department, F. J. Brakenridge, L.R.C.P., M.R.C.S. (extension), and P. J. A. Seccombe, M.A.Cantab, L.R.C.P., M.R.C.S.

### VACANCIES.

### Resident Medical Officers.

The following vacancies are announced this week, the amount of salary in each case being given after the name of the institution:—

Belgrave Hospital for Children, 79, Gloucester Street, S.W.—House Surgeon. Board, lodging, light, and fuel found. Applications to the Secretary by February 29th.

Birmingham Provident Dispensary (Sands Cox Trust, Hockley Branch).—Medical Officer. Minimum salary £250 per annum, with residence and gas. Applications to the Secretary, A. Derrington, 20, Weston Road, Handsworth, Birmingham, by March 2nd.

Bolton Infirmary and Dispensary.—Senior and Junior House Surgeons. Must have a registered medical and surgical qualification. Salary for the senior £120, for the junior £80 per annum, with apartments, board, and attendance. Applications and testimonials to Peter Kevan, Honorary Secretary, 12, Acresfield, Bolton, by March 3rd.

East London Hospital for Children, Glamis Road, Shadwell, E.—House Physician; must be duly qualified. Board, lodging, &c., provided, but no salary. Applications to Thomas Hayes, Secretary, by February 29th.

Manchester Royal Infirmary.—Clinical Assistant for the Barnes Convalescent Hospital, Cheadle. Appointment for six months. No salary, but board and lodging provided. Applications to the Chairman of the Medical Board, Royal Infirmary, Manchester, by February 29th.

Royal Free Hospital, Gray's Inn Road, W.C.—Resident Medical Officer (House Physician); doubly qualified. Appointment for six months, but eligible for re-election. No salary, but board, residence, and washing provided by the hospital. Applications to the Secretary by February 29th.

Sheffield Royal Hospital.—House Surgeon; unmarried. Salary 100 guineas per annum, with board (exclusive of wine and beer) and lodging. Appointment for two years. Applications to Dr. Sinclair White, Secretary to the Honorary Medical Staff, by February 29th.

St. Peter's Hospital, Henrietta Street, Covent Garden, W.C.—House Surgeon. Appointment for six months, but eligible for re-election. Salary at the rate of £100 per annum, with board, lodging, and washing. Applications to Irwin H. Beattie, Secretary, by March 2nd.

### Non-Resident Medical Officers.

Haverstock Hill and Malden Road Provident Dispensary, 132, Malden Road, N.W.—Medical Officer. Applications to the Honorary Secretary, Mr. G. G. Browne, by March 2nd.

Hospital for Women, Soho Square.—Assistant House Physician. Appointment for three months. Candidates must be fully qualified. Applications and testimonials to David Cannon, Secretary, before March 3rd.

Owens College, Manchester.—Junior Demonstratorship in Physiology and Histology. Salary £100 per annum. Applications to the Registrar by March 2nd.

University College, London.—Professorship of Pathology. Applications to the Secretary, J. M. Horsburg, M.A., by February 29th.

### BURDETT'S HOSPITALS AND CHARITIES:

**Being the Year-Book of Philanthropy, 1895.**

Edited by HENRY C. BURDETT,

*(The 1896 Edition is in the press, and will be published shortly.)*

London: The Scientific Press (Limited), 428, Strand, W.C.

Just Published. Third Edition. Re-written and much enlarged. Illustrated with nearly 50 Plans (mostly new ones), Diagrams, &c. Crown 8vo. Cloth gilt. Price 10s. 6d.

### COTTAGE HOSPITALS:

GENERAL, FEVER, AND CONVALESCENT.

**Their Progress, Management, and Work in Great Britain and Ireland, and the United States of America.**

With an Alphabetical List of Every Cottage Hospital at Present Opened.

BY HENRY C. BURDETT,

Author of "Hospitals and Asylums of the World," "Burdett's Hospitals and Charities," &c., &c.

This new Edition is issued in response to the great demand which has existed for the book during the past few years, and the wide interest it has excited amongst many classes of readers, both in this country and the United States. The Second Edition was published more than 15 years ago.

"An invaluable book of information on a subject which attracts increasing attention. . . . . A complete manual of all that relates to the founding, the cost, and the management of cottage hospitals. It embodies the results of wide experience, and it demonstrates the methods by which efficiency and economy may be secured. . . . . . No more complete manual could be required. It is a pleasure to see the care that has been bestowed on the work, and to bear testimony to its utility; few half-guineas could be better spent than in its purchase."—*St. James's Gazette.*

"Invaluable to all who are concerned in the management or contemplate the establishment of a cottage hospital."—*Truth.*

Now Ready. Price 21s. net.

**Volume V. of**

### THE JOHNS HOPKINS HOSPITAL REPORTS.

Its contents are as follows:—

The Malarial Fevers of Baltimore. By W. S. Thayer, M.D., and J. Hewetson, M.D.
Study of Some Fatal Cases of Malaria. By Lewellys F. Barker, M.D.
Studies in Typhoid Fever. By William Osler and others.

London: THE SCIENTIFIC PRESS (Limited), 428, Strand, W.C.

### BLACKIE AND SON'S ELEMENTARY TEXT-BOOKS.

*FULLY ILLUSTRATED.*

**ELEMENTARY PHYSIOLOGY.** By Professor AINSWORTH DAVIS. With Appendix for Agricultural Students. Cloth, 2s.

"Written in a clear, forcible style, and crammed with detailed information which is arranged with great method. The illustrations are profuse and admirable, and the directions given to students on practical work are excellent."—*British Med. Journal.*

**AN ELEMENTARY TEXT-BOOK OF PHYSIOLOGY.** By J. M'GREGOR ROBERTSON, M.A., M.B., Lecturer in Physiology, Queen Margaret College. New and Revised Edition. Cloth, 4s.

"A good system of arrangement and clear expressive exposition distinguish this book. Definitions of terms are remarkably lucid and exact."—*Saturday Review.*

**ELEMENTARY PHYSIOLOGY.** By VINCENT T. MURCHE. With Coloured Illustrations. Cloth, 2s.

"Its teaching is sound, concise, and up to date, and the care with which the little book is illustrated leaves little to be desired either by teacher or pupil."—*Hospital Gazette.*

**AN ELEMENTARY TEXT-BOOK OF ANATOMY.** By HENRY EDWARD CLARK, Fellow of the Faculty of Physicians and Surgeons of Glasgow. Cloth, 5s.

"The author's experience as a teacher and his reputation as an anatomist are a sufficient guarantee for the general accuracy of its contents. It is methodically arranged, clearly written, and well illustrated."—*Edinburgh Medical Journal.*

**ELEMENTARY HYGIENE.** By H. ROWLAND WAKEFIELD. Cloth, 2s.

"Contains a large amount of information, conveyed in clear and precise terms."—*British Medical Journal.*

London: BLACKIE and SON (Limited), Old Bailey.

### "THE HOSPITAL"

AN INDEPENDENT JOURNAL OF
**The Medical Sciences**
AND
**Hospital Administration,**
WITH WHICH IS ISSUED WEEKLY
A SPECIAL
**SUPPLEMENT FOR NURSES.**

PUBLISHED EVERY SATURDAY.
PRICE TWOPENCE.

Matrons, Sisters, or Nurses will find in THE HOSPITAL a chronicle of the latest Nursing News, Practical Articles of the highest value to those who wish to keep themselves abreast with the times, and brightly written articles from Nurses all over the world.

SUBSCRIPTIONS CAN COMMENCE AT ANY TIME.

### TERMS OF SUBSCRIPTION.

### WEEKLY ISSUE.

| | Great Britain. | Foreign and Colonial. |
|---|---|---|
| For One Year ... ... | 10s. 6d. ... | 15s. 2d. |
| For Six Months .... | 5s. 3d. ... | 7s. 7d. |
| For Three Months... | 2s. 8d. ... | 3s. 10d. |

### MONTHLY PARTS.

| | Post Free to any Part of the World. |
|---|---|
| For One Year ... ... | 8s. 6d. |
| For Six Months ... ... | 4s. 3d. |
| For Three Months ... ... | 2s. 2d. |

Postal Orders and Cheques should be made payable to "The Scientific Press, Limited."

N.B.—In order to prevent delay, all business communications should be addressed to "THE MANAGER" only, and not to any person individually.

### CHANGE OF ADDRESS.

In event of change of address, notice should be forwarded to the Publishers by the Saturday previous to date of publication, and the old address should also be given.

"THE HOSPITAL" can be obtained of all Booksellers and Newsagents in the United Kingdom, and at all the Railway Stalls, of Messrs. W. H. Smith and Son; and also from the following Agents in the Colonies and Abroad:—

PARIS: Librairie Galignani, 224, Rue de Rivoli.
BERLIN: Messrs. A. Asher and Co.
LEIPSIG: Mr. F. A. Brockhaus.
NEW YORK: The International News Co.
MONTREAL: The Montreal News Co.
TORONTO: The Toronto News Co.
MELBOURNE, SYDNEY, ADELAIDE, and ZEALAND: Messrs. Gordon and Gotch.
INDIA: At all the Railway Bookstalls and Agencies of Messrs. A. H. Wheeler and Co.
SOUTH AFRICA: Messrs. Juta, Cape Town.

PUBLISHING OFFICES: 428, STRAND, LONDON, W.C

### STANDARD TEXT-BOOKS.

Just Out. Second Edition.

**INFANT FEEDING BY ARTIFICIAL MEANS:** A Scientific and Practical Treatise on the Dietetics of Infancy. By S. H. SADLER, Author of "Suggestions to Mothers," "Management of Children," "Education." Second Edition, with a new chapter, and several coloured plates, illustrative of feeding-bottles used in ancient times, &c. Crown 8vo., cloth gilt, with 10 plates and many Illustrations in the text, price 5s. Facsimile Autograph Letters from the late Sir Andrew Clark, M. Pasteur, &c., &c.

"Mrs. Sadler's book deals with the question of the artificial feeding of infants, and contains a very useful collection of the views of the best-known English authorities upon the subject. The truly terrible ignorance displayed by mothers, especially amongst the poor, upon the subject of infant feeding is answerable for an infant mortality so great as to be appalling."—*Daily Chronicle.*

**DIET IN SICKNESS AND IN HEALTH.** By MRS. ERNEST HART, formerly Student of the Faculty of Medicine of Paris, and of the London School of Medicine for Women. Demy 8vo, blue buckram, gilt, with numerous illustrations, price 3s. 6d. With an introduction by Sir Henry Thompson, F.R.C.S., M.B. Lond., who says: —"I do not hesitate to express my opinion that the present volume forms a handbook to the subject thus briefly set forth in these few lines, which will not only interest the dietetic student, but offer him, within its modest compass, a more complete epitome thereof than any work which has yet come under my notice."

**A PRACTICAL HANDBOOK OF MIDWIFERY.** By FRANCIS W. N. HAULTAIN, M.D. A practical manual produced in a portable and convenient form for reference, and especially recommended for its compactness, conciseness, and clearness. 18mo., profusely illustrated with original cuts, tables, &c.; about 260 pp., handsomely bound in leather. Price 6s.

"One of the best of its kind, and well fitted to perform the functions its author claims for it."—*Edinburgh Medical Journal.*

**THE ART OF MASSAGE.** By A. CREIGHTON HALE. A complete Text-book of this valuable art. The most practical, simple, and generally useful work published on the subject. With fully illustrated chapters on the Anatomy of the Human Body. Demy 8vo, nearly 200 pp., cloth gilt, profusely illustrated with over 70 special cuts. Price 6s.

"The latest and most complete book we know on Massage."—*Queen.*

**NURSING: ITS THEORY AND PRACTICE.** Being a complete Text-Book of Medical, Surgical, and Monthly Nursing. By PERCY G. LEWIS, M.D., M.R.C.S., L.S.A. Always a standard work on Nursing, it now occupies the position of a classic, being used throughout the world in Training Schools and by Private Students. Eighth Edition. Greatly enlarged and thoroughly revised. Profusely Illustrated. Crown 8vo, cloth, gilt. Price 3s. 6d.

"We warmly recommend it as a nursing manual . . . full of useful hints and information."—*Birmingham Medical Review.*

"Full of interest and instruction . . . cannot fail to assist nurses in their work."—*British Medical Journal.*

**SURGICAL WARD-WORK AND NURSING.** By ALEXANDER MILES, M.D. (Edin.), C.M., F.R.C.S.E. A practical Manual of Clinical Instruction for Nurses and Students. This book will be found especially useful to beginners, to whom its simple description of elementary principles and treatment in surgery will render it invaluable. Demy 8vo, 200 pp., cloth boards, copiously illustrated. Price 3s. 6d.

"Will furnish much-needed guidance to the student and the nurse in the early stages of their apprenticeship."—*Times.*

**NURSING BOOKS AT FULL DISCOUNT.**
*LIST POST FREE.*
The Theory and Practice of Nursing (Percy Lewis, M.D.), 3/6 for **2/8.**
The Nurse's Dictionary (Honnor Morten), 2/- for **1/6.**
The Nurse's Case Book, 6d. for **3½d.**

### ROYAL NATIONAL PENSION FUND FOR NURSES.

*President*—**H.R.H. THE PRINCESS OF WALES.**

*Chairman*—WALTER H. BURNS, Esq. *Deputy-Chairman*—HENRY C. BURDETT, Esq.

**PENSIONS.** **SICKNESS.** **ACCIDENT.**

### Total Invested Funds, £250,000.

Nurses are invited to join the Fund on account of the substantial and exceptional advantages which it offers them and which *they cannot obtain elsewhere.* The following are the chief points:—

1. **The Fund is Mutual and essentially Co-operative.**
2. **Easy Payment of Premiums.**
   Nurses can pay their premiums monthly or otherwise as best suits their convenience.
3. **The Fund is open to every Nurse.**
   Nurses can assure for Pensions of any amount commencing at any age.
4. **An Investment and Savings Bank.**
   Those entering under the returnable scale can have their premiums returned to them with compound interest, less a small deduction for working expenses. Interest allowed on deposits.
5. **Additions to Pensions.**
   Besides the Pension entered for, substantial additions thereto may be anticipated in the shape of bonuses.
6. **Sickness and Accident Assurance.**
   Policies are issued in connection with Pension policies assuring 10s., 15s., or 20s. a week in cases of incapacity from work through sickness or accident.

**Full particulars to be obtained from THE SECRETARY,**

ROYAL NATIONAL PENSION FUND FOR NURSES,

28, FINSBURY PAVEMENT, LONDON, E.C.

